# Serial imaging using [18F]Fluorodeoxyglucose positron emission tomography and histopathologic assessment in predicting survival in a population of surgically resectable distal oesophageal and gastric adenocarcinoma following neoadjuvant therapy

**DOI:** 10.1007/s12149-017-1159-2

**Published:** 2017-03-15

**Authors:** Varun Manoharan, Soon Lee, Shanley Chong, June Yap, Nick Coupe, Robert Wilson, Neil Merrett, Weng Ng, Michael Lin

**Affiliations:** 1 University of New South Wales, Liverpool Hospital, Sydney, NSW 2170 Australia; 2 University of Western Sydney, Liverpool Hospital, Sydney, NSW 2170 Australia; 3 Department of Anatomical Pathology, Liverpool Hospital, Sydney, NSW 2170 Australia; 4 Department of Nuclear Medicine and PET, Ground Floor, New Clinical Building, 1 Elizabeth Drive, Liverpool Hospital, Sydney, NSW 2170 Australia; 5 Department of Medical Oncology, Liverpool Hospital, Sydney, NSW 2170 Australia; 6 Department of Surgery, Liverpool Hospital, Sydney, NSW 2170 Australia

**Keywords:** Oesophageal cancer, Gastric cancer, Treatment response, FDG, PET, PET-CT

## Abstract

**Background and objectives:**

We retrospectively evaluated the value of PET/CT in predicting survival and histopathological tumour-response in patients with distal oesophageal and gastric adenocarcinoma following neoadjuvant treatment.

**Methods:**

Twenty-one patients with resectable distal oesophageal adenocarcinoma and 14 with gastric adenocarcinoma between January 2002 and December 2011, who had undergone serial PET before and after neoadjuvant therapy followed by surgery, were enrolled. Maximum standard uptake value (SUVmax) and metabolic tumour volume were measured and correlated with tumour regression grade and survival.

**Results:**

Histopathological tumour response (PR) is a stronger predictor of overall and disease-free survival compared to metabolic response. ∆%SUVmax ≥70% was the only PET metric that predicted PR (82.4% sensitivity, 61.5% specificity, *p* = 0.047). Histopathological non-responders had a higher risk of death (HR 8.461, *p* = 0.001) and recurrence (HR 6.385, *p* = 0.002) and similarly in metabolic non-responders for death (HR 2.956, *p* = 0.063) and recurrence (HR 3.614, *p* = 0.028). Ordinalised ∆%SUVmax showed a predictive trend for OS and DFS, but failed to achieve statistical significance.

**Conclusions:**

PR was a stronger predictor of survival than metabolic response. ∆%SUVmax ≥70% was the best biomarker on PET that predicted PR and survival in oesophageal and gastric adenocarcinoma. Ordinalisation of ∆%SUVmax was not helpful in predicting primary outcomes.

## Introduction

Despite the increase in the incidence of oesophageal adenocarcinoma since 1988 and the steady decline in the incidence of gastric adenocarcinoma since 1982, oesophageal and gastric malignancies collectively accounted for 2317 deaths in Australia in 2010 [1]. While surgery remains the mainstay of curative treatment, the administration of neoadjuvant multimodality therapy has been shown to increase rates of histopathological complete response and entail modest survival benefit over surgery alone [2]. Positron emission tomography (PET) using ^18^F-2-fluoro-2-deoxyglucose (^18^F-FDG) permits in vivo characterisation of pathological ^18^F-FDG retention and PET has gained acceptance for initial staging of oesophageal malignancy by improving the detection of occult distant metastases [3]. Recently, several authors have investigated the predictive value of PET metrics for early response to therapy in oesophageal and gastric neoplasms, given that PET has been shown to be useful in other cancers [4–7].

Tumour regression grades (TRG), a measure of histopathological tumour response (PR) based on an estimation of the percentage of residual tumour tissue in relation to the macroscopically identifiable tumour bed at the primary site, was adopted from studies conducted on gastric cancers [8] and oesophageal adenocarcinomas [9]. Lordick, et al. [10] validated the use of therapy-induced changes in PET metrics to predict PR and to stratify distal oesophageal and gastric cardia adenocarcinoma patients into different prognostic groups. They concluded that 35% regression of maximum standardised uptake value (SUV_max_), a semi-quantitative measure of ^18^F-FDG retention in the primary tumour bed was the optimal cut-off to identify histopathological responders with 100% sensitivity and 58% specificity. However, the evidence regarding the predictive value of PET-based biomarkers in gastric cancers is limited and inconsistent. One prospective study on advanced gastric cancer patients confirmed the highly predictive value of 35% reduction in SUV_max_ on PR with 77% sensitivity and 86% specificity [11], while one small study comprising locally advanced gastric cancers showed a 45% decrease in SUV from baseline and day 35 significantly predicted PR whereas the change at day 15 did not [12].

There is increasing interest in investigating the prognostic value of PET volumetric parameters such as metabolic tumour volume (MTV), but the utility of this more novel imaging biomarker is experimental. One study reported that a 63% reduction in MTV was the optimal cut-off to identify histopathologically responding distal oesophageal adenocarcinoma with 91% sensitivity and 90% specificity [13]. The evidence on the predictive value of MTV in gastric cancer is scarce.

Several authors have validated that metabolic responders (MR^+^) identified on PET had better prognosis [10, 11, 14]. Some studies have shown variable thresholds [15] in predicting PR and survival on PET, while others failed to validate the predictive power of PET metrics [16, 17]. Lee did not find a correlation between the change in SUV_max_ of the primary tumour with PR after neoadjuvant chemotherapy and surgery in gastric carcinoma [18]. Vallbohmer also did not find a correlation between a change in SUV_max_ (baseline and post-treatment PET at 2 weeks) and PR (<10% viable cells) or overall survival in oesophageal carcinoma [19]. This study aims to retrospectively evaluate the performance of PET in predicting survival and PR to neoadjuvant therapy in patients with surgically resected distal oesophageal and gastric adenocarcinoma.

## Method

### Patient population

**Fig. 1:**
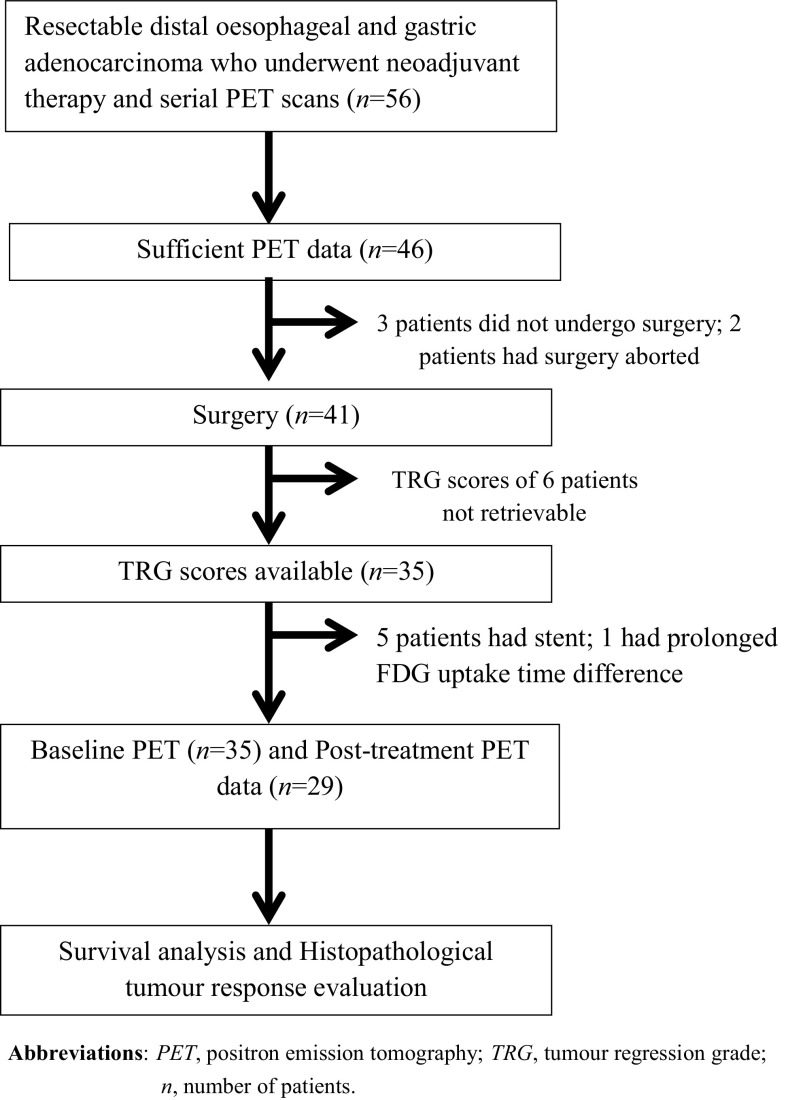
Patient selection. *PET* positron emission tomography, *TRG* tumour regression grade, *n* number of patients

Fifty-six patients with newly diagnosed resectable distal oesophageal and gastric adenocarcinoma who underwent neoadjuvant therapy and serial ^18^F-FDG PET scans at Liverpool Hospital (Sydney, Australia) between January 2002 and December 2011 were included. 35 patients (21 patients with distal oesophageal adenocarcinoma and 14 patients with gastric adenocarcinoma) with sufficient PET data and TRG scores were analysed (Fig. 1). Patients with non-resectable disease at diagnosis, non-FDG-avid primary tumour on initial PET scan (PET-1), insufficient PET data, active concurrent cancer unrelated to oesophageal and gastric cancer, previous neoadjuvant therapy prior to initial PET and those who did not undergo surgery were excluded. Post-treatment PET (PET-2) parameters were not evaluated in one patient with a significant difference in uptake time between the two PET scans (64 min) and in five patients who had oesophageal stents implanted after PET-1. Most patients underwent PET/CT scans apart from two patients where PET-alone imaging was performed.

Tumour staging was based on the sixth edition of the American Joint Committee of Cancer (AJCC) Staging Manual [20], but classification of tumour location was based on the seventh edition [21].

### Patient information

Demographic, clinical and follow-up data were gathered through various databases including the South Western Sydney Area Health Service’s (SWSAHS) online patient information system, Cancer Therapy Centre (MOSAIQ), Departments of Surgery and Nuclear Medicine and PET.

### PET imaging

All patients underwent a baseline PET scan for staging (PET-1) and a post-treatment PET scan (PET-2). PET-alone scans operating in three-dimensional mode (Allegro, Philips Medical Systems, Milpitas, CA, US) with germanium source attenuation were performed prior to February 2006, and PET/CT scans (Gemini GXL-6, Philips Medical Systems, Miltipas, CA, US) using low-dose CT without contrast enhancement for attenuation correction.

A standardised protocol comprised a minimum 4-h fasting period and blood glucose levels <10 mmol/L prior to ^18^F-FDG (5.14 MBq/kg), administration intravenously. Patients were scanned after an uptake period of approximately 60 min.

### PET data analysis

PET scans were analysed by two accredited Nuclear Medicine physicians in consensus (M.L. and J.Y.) according to a standardised protocol where the SUV_max_ was measured using a 15 mm wide region of interest around the primary tumour. MTV was measured using vendor’s software with a SUV_max_ threshold that best delineated the tumour. Scans (PET-1 and PET-2) were analysed blinded from all clinical, pathological and imaging data apart from the knowledge that all patients had oesophageal and gastric malignancy and had completed neoadjuvant therapy. PET-1 and PET-2 measurements and any absolute and relative differences in ^18^F-FDG uptake were correlated with TRG and survival. If no residual tumour was visible and uptake was indistinguishable from background oesophageal or gastric activity on post-treatment scan, no volumetric measurement was attempted and the percentage reduction in abnormal tracer uptake is assigned 100%. Metabolic responders (MR^+^) are patients with ∆%SUV_max_ ≥70%.

### Histopathological response evaluation

Surgical specimens were retrospectively examined by a single pathologist (S.L.). TRG score was assessed semi-quantitatively into either complete (TRG 1a: no residual tumour), subtotal (TRG 1b: <10% of residual tumour), partial (TRG 2: 10–50% of residual tumour) and minimal response (TRG 3: >50% residual tumour) based on Becker et al. [8]. The pathologist was blinded from all clinical, pathological and imaging data. Patients with complete or subtotal tumour regression were classified as histopathological responders (PR^+^). All other patients were classified as non-responders (PR^−^).

### Follow-up

Disease-status and survival status at the time of census were recorded. Overall survival (OS) was calculated from the date of PET-1 to date of death or date of most recent follow-up. Disease-free survival (DFS) was calculated from date of surgery to the date of confirmed recurrence. If death was a direct consequence of surgery within 2 weeks of surgery, then the patient was excluded from survival analyses.

### Statistical analysis

Absolute numbers and percentages were computed to describe the patient population, and quantitative values are expressed as median and range. Chi-square test was used to examine associations between categorical variables. Receiver operator characteristics (ROC) curve was performed to find the optimal cut-offs of the PET parameters. Survival curves were generated using Kaplan–Meier estimates and significance of difference between curves was tested with log-rank tests. Univariate analysis of survival was performed using Cox regression analysis and the estimated hazard ratio (HR) and 95% confidence interval (CI) were reported. All statistical analyses were performed using IBM SPSS Statistics 21 and *p* < 0.05 was considered statistically significant.

## Results

### Patient characteristics

There was a preponderance of male subjects and neoadjuvant chemotherapy-alone treatment in the cohort (Table 1). Most patients were node negative on staging PET.

**Table 1:** Patient characteristics and clinicopathological parameters

Parameters	*n* (%)
Gender	
Males	30 (85.7)
Females	5 (14.3)
Median age at diagnosis	61.7 years (40.6–74.5)
Histology	
Signet ring cell	4 (11.4)
Non-Signet ring cell	31 (88.6)
Tumour Location (AJCC 7th ed.)	
Distal oesophagus	18 (51.4)
Stomach	17 (48.6)
Cardia of stomach	9
Fundus	1
Body of stomach	4
Antrum	3
Pylorus	0
Tumour Grade	
Moderate	17 (48.6)
Poor	18 (51.4)
Clinical T stage at diagnosis (AJCC 6th ed.)	
T_2_	3 (8.6)
T_3_	23 (65.7)
T_4_	6 (17.1)
Missing	3 (8.6)
Nodal involvement on staging PET	
N^−^	26 (74.3)
N^+^	9 (25.7)
Overall clinical stage at diagnosis (AJCC 6th ed.)	
I	2 (5.7)
II	12 (48.6)
III	20 (55.1)
Missing	1 (2.9)
Type of neoadjuvant therapy	
CT alone	20 (57.1)
CRT	9 (25.7)
RT alone	3 (8.6)
Not specified	3 (8.6)
TRG	
1a	3 (8.6)
1b	13 (37.1)
2	4 (11.4)
3	15 (42.9)
Recurrence	
No	18 (51.4)
Yes	17 (48.6)
Median disease-free survival	13.8 months (2.1-122.4)
Dead	
No	15 (42.9)
Yes	20 (57.1)
Median overall survival	22.3 months (9.6-122.4)
Median follow-up period	22.7 months (10.8-122.4)
Two-year overall survival rate	40.0%

*T stage*, depth of invasion, *N stage* nodal involvement, *PET* positron emission tomography, *AJCC* American Joint Committee of Cancer Staging Manual, *N* ^−^ negative nodal involvement, *N* ^+^ positive nodal involvement; *CT* chemotherapy, *CRT* chemoradiotherapy, *RT* radiotherapy, *TRG* tumour regression grade

There was a significant difference (*p* = 0.002) in the median patient weight at PET-1 (79 kg, 48–115) compared to PET-2 of (76 kg, 55–118). Median uptake time at PET-1 was 63 min and for PET-2 was 64 min (*p* = 0.878) with a median difference in uptake times of 6 min (0–22). The median interval between the two PET scans was 106 days (55–153).

### PET and histopathological tumour response evaluation

The relative reduction in SUV_max_ (∆%SUV_max_) ≥70% was the only optimum cut-off to predict PR (*p* = 0.047) on ROC analysis with 82.4% sensitivity and 61.5% specificity. There was a trend for a greater proportion of PR^+^ having an absolute reduction in SUV_max_ (∆SUV_max_) ≥5.75 (*p* = 0.071). Other PET metrics did not retain statistical significance and were dichotomised at the respective median values (Table 2). By ordinalising ∆%SUV_max_ into a schema similar to that of TRG with complete response (∆%SUV_max_ = 100%), subtotal response (∆%SUV_max_ ≥70%), partial response (∆%SUV_max_ ≥35%) and minimal response (∆%SUV_max_ <35%), no significant result was attained.

**Table 2:** Predictive value of various PET parameters of tumour regression grade (TRG) score

	*n* (%)		Chi-square	*p*
	TRG1a–1b	TRG2–3		
Baseline SUV_max_				
SUV1_max_ ≥9.70	10 (28.6)	10 (28.6)	0.345	0.557
SUV1_max_ <9.70	6 (17.1)	9 (25.7)		
Post-treatment SUV_max_				
SUV2_max_ ≥3.75	5 (17.2)	8 (27.6)	0.386	0.534
SUV2_max_ <3.75	8 (27.6)	8 (27.6)		
Metabolic response based on ∆SUV_max_				
∆SUV_max_ ≥5.75	10 (34.5)	7 (24.2)	3.254	0.071
∆SUV_max_ <5.75	3 (10.3)	9 (31.0)		
Metabolic response based on ROC analysis of ∆%SUV_max_				
∆%SUV_max_ ≥70%	8 (27.6)	4 (13.8)	3.948	**0.047**
∆%SUV_max_ <70%	5 (17.2)	12 (41.4)		
Baseline MTV (cm^3^)				
MTV1 ≥47.30	8 (27.6)	10 (34.5)	0.024	1.000
MTV1 <47.30	8 (27.6)	9 (31.0)		
Post-treatment MTV (cm^3^)				
MTV2 ≥12.00	3 (11.0)	7 (22.8)	1.008	0.315
MTV2 <12.00	8 (27.6)	8 (27.6)		
Missing	3 (11.0)			
Metabolic response based on ∆MTV				
∆MTV ≥39.40	7 (22.8)	9 (31.0)	0.035	0.851
∆MTV <39.40	4 (13.2)	6 (22.0)		
Missing	3 (11.0)			
Metabolic response based on ∆% MTV				
∆%MTV ≥80%	5 (14.0)	6 (22.0)	0.077	0.781
∆%MTV <80%	6 (22.0)	9 (31.0)		
Missing	3 (11.0)			
Metabolic response based on various cut-offs of ∆%SUV_max_				
∆%SUV_max_ = 100%	2 (6.9)	2 (6.9)	3.883	0.274
∆%SUV_max_ ≥70–99%	6 (20.7)	2 (6.9)		
∆%SUV_max_ ≥35–69%	5 (17.2)	9 (31.1)		
∆%SUV_max_ <35%	0 (0.0)	3 (10.3)		

Statistical significant result is in bold

Post-treatment scans in 5 patients were excluded from analysis due to oesophageal stent insertion. One patient was excluded due to significant difference in uptake times between the two scans

*PET* positron emission tomography, *TRG* tumour regression grade, *SUV1*_*max*_ baseline SUV_max_, *SUV2*_*max*_ post-treatment SUV_max_, *∆SUV*_*max*_ absolute reduction in SUV_max_, *∆%SUV*_*max*_ relative reduction in SUV_max_, *MTV *metabolic tumour volume, MTV1 baseline MTV, *MTV2* post-treatment MTV, *∆MTV* absolute reduction in MTV, *∆%MTV* relative reduction in MTV

### PET, TRG and survival analysis

**Fig. 2:**
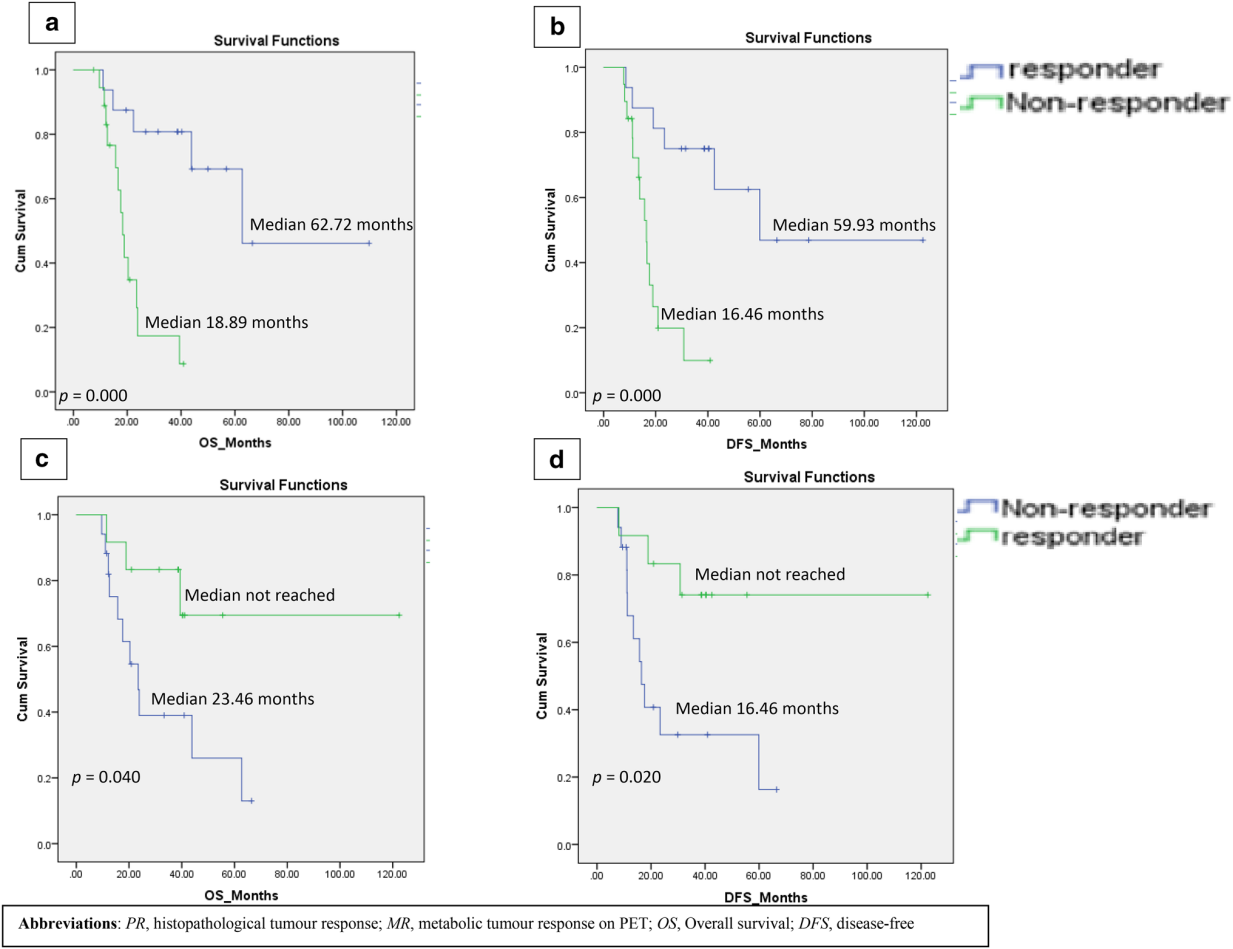
Kaplan–Meier Analysis. PR and OS (**a**), PR and DFS (**b**), MR and OS (**c**), MR and DFS (**d**). *PR* histopathological tumour response, *MR* metabolic tumour response on PET, *OS* overall survival, *DFS* disease-free

MR^+^ and PR^+^ had a significantly longer OS and DFS than their non-responding counterparts (Fig. 2). Median OS and DFS were not reached in MR^+^.

PR^−^ had a significantly greater risk of death (HR 8.461; *p* = 0.001) and recurrence (HR 6.385; *p* = 0.002) (Table 3). Similarly, metabolic non-responders (MR^−^) with ∆%SUV_max_ <70% entailed a significantly greater risk of death (HR 2.956, *p* = 0.063) and recurrence (HR 3.614; *p* = 0.028) (Table 3). PR was a stronger predictor of OS (HR 8.461 vs 2.956) and DFS (HR 6.385 vs 3.614) compared to ∆%SUV_max_≥70%.

**Table 3:** Prognostic value of metabolic parameters on PET and histopathological tumour response for survival

	*n* (%)	Univariate Cox regression test			Univariate Cox regression test		
		OS HR	95% CI	*p*	DFS HR	95% CI	*p*
Baseline SUV_max_							
SUV1_max_ ≥9.70	20 (57.1)	1.000		**0.044**	1.000		**0.030**
SUV1_max_ < 9.70	15 (42.9)	2.589	1.025–6.535		2.797	1.107–7.071	
Post-treatment SUV_max_							
SUV2_max_ ≥3.75	13 (44.8)	1.000		0.376	1.000		0.352
SUV2_max_ < 3.75	16 (55.2)	1.627	0.554–4.780		1.658	0.572–4.812	
Metabolic response based on ∆SUV_max_							
∆SUV_max_ ≥ 5.75	17 (58.6)	1.000		0.112	1.000		0.066
∆SUV_max_ < 5.75	12 (41.2)	2.223	0.829–6.013		2.563	0.941–6.981	
Metabolic response based on ROC analysis of ∆%SUV_max_							
∆%SUV_max_ ≥70%	12 (41.2)	1.000		0.063	1.000		**0.028**
∆%SUV_max_ <70%	17 (58.6)	2.956	0.945–9.247		3.614	1.150–11.533	
Baseline MTV (cm^3^)							
MTV1 ≥47.30	18 (51.4)	1.000		0.141	1.000		0.133
MTV1 < 47.30	17 (48.6)	1.996	0.795–5.013		2.031	0.806–5.118	
Post-treatment MTV (cm^3^)							
MTV2 ≥12.00	10 (34.5)	1.000		0.817	1.000		0.795
MTV2 < 12.00	16 (55.2)	0.860	0.241–3.074		0.845	0.238–3.003	
Missing	3 (10.3)						
Metabolic response based on ∆MTV							
∆MTV ≥39.40	16 (55.2)	1.000		0.305	1.000		0. 362
∆MTV < 39.40	10 (34.5)	1.182	0.583–5.639		1.698	0.544–5.296	
Missing	3 (10.3)						
Metabolic response based on ∆% MTV							
∆%MTV ≥80%	10 (34.5)	1.000		0.707	1.000		0. 681
∆%MTV < 80%	16 (55.2)	0.792	0.235–2.672		0.775	0.231–2.605	
Missing	3 (10.3)						
Metabolic response based on various cut-offs of ∆%SUV_max_							
∆%SUV_max_ = 100%	4 (13.8)	1.000			1.000		
∆%SUV_max_ ≥70–99%	8 (27.6)	0.402	0.056–2.898	0.366	0.454	0.063–3.247	0.431
∆%SUV_max_ ≥35–69%	14 (48.3)	1.552	0.331–7.727	0.577	2.137	0.460–9.922	0.332
∆%SUV_max_ <35%	3 (10.3)	3.241	0.429–24.495	0.254	3.718	0.491–28.183	0.204
Histopathological tumour response							
TRG_1a–1b_	16 (45.7)	1.000			1.000		
TRG_2–3_	19 (54.3)	8.461	2.355–30.396	0.001	6.385	2.019–20.195	0.002

Statistical significant result is in bold

Post-treatment scans in five patients were excluded from analysis due to oesophageal stent insertion. One patient was excluded due to significant difference in uptake times between the two scans

*PET* positron emission tomography, *OS* overall survival, *DFS* Disease-free survival, *HR* Hazard ratio, *CI* confidence interval, *n* number of patients, *SUV1*_*max*_ baseline SUV_max_, *SUV2*_*max*_ post-treatment SUV_max_, *∆SUV*_*max*_ absolute reduction in SUV_max_, *∆%SUV*_*max*_ relative reduction in SUV_max_, *MTV *metabolic tumour volume, *MTV1* Baseline MTV, *MTV2* post-treatment MTV, *∆MTV* absolute reduction in MTV, *∆%MTV* relative reduction in MTV, *TRG* tumour regression grade

For PET-1, baseline SUV_max_ <9.70 was the only statistically significant predictor of poor OS (HR 2.589, *p* = 0.044). The ∆%SUV_max_ <70% cut-off showed a trend (*p* = 0.063) towards poorer OS. Both groups reported a higher risk of recurrence (*p* = 0.030; *p* = 0.028) (Table 3). All other PET metrics were not statistically significant.

When we analysed TRG as a binary parameter-complete or subtotal (TRG 1a and TRG 1b), and partial or minimal response (TRG 2 and 3), TRG was significantly associated with survival status (*p* = 0.002) on Chi-square analysis.

## Discussion

The correlations between TRG, survival and PET-metrics have not been widely reported in oesophageal and gastric adenocarcinomas. This study comprised a heterogeneous group with resectable adenocarcinoma of the distal oesophagus and stomach. Although surgical treatment of oesophageal and gastric cancers occurs in relatively low volume centres in Australia, we have equivalent short and long term outcomes compared to Asian and European institutions [22, 23]. Therefore, our findings on the predictive performance of PET for survival and PR in this group are applicable to other populations. The study is also strengthened with centralized PET scan interpretation in consensus.

We found that a favourable metabolic response is associated with favourable PR. A reduction of SUV_max_ of the primary ≥70% following neoadjuvant treatment was associated with a TRG_1a–1b_. Our study showed that ∆%SUV_max_ ≥70% (*p* = 0.047) was the optimal cut-off that characterised PR with 82.4% sensitivity and 61.5% specificity. To our knowledge, the association between PR and PET metrics have not been studied extensively in gastric cancers. This is in line with a small study of 35 patients using PET-alone system which demonstrated ∆%SUV_max_ ≥35% predicted survival in locally advanced gastric cancer [11]. Our data included only two patients who underwent PET-alone imaging and while we acknowledge PET-alone and PET-CT can produce differences in SUV due to different methods of attenuation correction, the difference is reported to be relatively small in the clinical setting for non-osseous lesions of a sufficient size (our cohort comprised mostly T3 or T4 tumours) [24, 25].

Although there was a trend for an absolute therapy-induced change in SUV_max_ (*p* = 0.07) in predicting PR, we showed relative reduction as a stronger predictor. This may be due to the variable physiologic background FDG uptake in the oesophagus and stomach and relative reduction appears to be a better metric to gauge response. There is currently no consensus on the optimal thresholds in determining metabolic response on PET and various investigators have found different cut-offs [10, 17, 26, 27]. This most likely reflects different camera specifications and methodology and a standardised protocol in future prospective trials is mandatory [28].

There are conflicting results regarding the use of PET metrics in predicting survival in gastric cancer [29–31]. Our results demonstrated both MR^+^ and PR^+^ had significantly longer OS and DFS than non-responders (Fig. 2). Becker et al. [8] showed that PR^+^ (TRG 1a/1b) in 480 gastric adenocarcinoma patients had a significantly longer OS (128.6 months) compared to partial (61.9 months) and minimal (40.1 months) responders and similar conclusions were drawn from studies on distal oesophageal adenocarcinoma [9]. In our study, MR^+^ had a significantly longer OS (median NR vs 23.5 months) and longer DFS (median NR vs 16.5 months). These findings are similar to those from the MUNICON I trial [10]. There was a trend for MR^−^ to have an almost threefold increase in risk of death (*p* = 0.06) and a significantly shorter DFS (*p* = 0.028) (Table 3). Although high SUV_max_ in many tumours have been shown to be poor prognosticators [32], in our study a high baseline SUV_max_ (>9.7) did not predict poor OS. This observation has also been shown in one study where patients with metastatic disease had lower SUV_max_ and SUV_mean_ compared to M0 patients [33] and in another, no significant difference was found between limited and disseminated gastric cancers [34]. This may be due to several poor prognostic histological sub-types having low glucose metabolism, e.g. signet-ring cell or mucinous adenocarcinoma. Recent evidence also suggests a relationship between FDG avidity and HER2 expression and PET may have the potential to predict tumour phenotype [35].

PR^+^ in our study had a greater reduction in tumour ^18^F-FDG metabolism, highlighting the potential to formulate PET-guided treatment algorithm, which has been validated in the MUNICON II trial [36]. The greater histopathological remission rate among MR^−^ of the MUNICON II trial compared to those in the MUNICON I trial was attributed to the PET-based early metabolic assessment and subsequent escalation of therapy in MR^−^ from chemotherapy-alone to chemoradiotherapy [10, 36]. MR^−^ in the MUNICON I trial had their chemotherapy stopped after 2 weeks and went directly to surgical resection potentially avoiding toxicity from futile chemotherapy. Despite the addition of radiotherapy to cisplatin or 5-fluorouracil based chemotherapy in MUNICON II trial, MR^−^ still had a poor prognosis. A recent trial comprising a small number of patients suggested that changing chemotherapy regimens (to taxane-based) in PET non-responding patients may improve outcomes [37].

To our knowledge, this is the first study to ordinalise ∆%SUV_max_ similar to TRG scores using ∆%SUV_max_ ≥70% and ∆%SUV_max_ ≥35% cut-offs. Although it failed to achieve statistical significance, our analysis showed a predictive trend for DFS and OS (Table 3). This warrants further investigation in a larger cohort.

Volumetric measurements on PET is emerging as an important novel imaging biomarker in predicting prognosis in non-small cell lung (NSCLC) [7] and oesophageal cancers [13, 38], but there is limited evidence in gastric malignancies. In our study, neither the median MTV nor the MTV determined by ROC analysis (data not shown) predicted PR and survival, perhaps due to the difficulty and inaccuracy in delineating MTV using different SUV thresholds. This may partly be due to the variable physiological ^18^F-FDG uptake in gastric mucosa which attenuates the tumour to background ratio. Hence, MTV may not have the same prognostic value in gastric cancer compared with NSCLC or oesophageal cancer.

This retrospective study comprised only FDG-avid gastric tumours. It is well known that a significant proportion of gastric cancers can be falsely negative on FDG PET in particular in tumours rich in mucin and our data is not applicable to all gastric cancers [39–41]. We combined patients with distal oesophageal and gastric cancers due to small sample size and a larger population could have allowed subgroup analyses.

In conclusion, PR was a stronger prognostic indicator than metabolic response, and ∆%SUV_max_ was the best PET-based biomarker that predicted PR and survival in oesophageal and gastric adenocarcinoma. This study highlighted the potential role of PET in optimising treatment protocols and allows non-responders to be detected early to have escalation of treatment in this poor prognostic group.
